# Behavioural innovation and cultural transmission of communication signal in black howler monkeys

**DOI:** 10.1038/srep13400

**Published:** 2015-08-25

**Authors:** M. Briseño-Jaramillo, A. Estrada, A. Lemasson

**Affiliations:** 1 Universidad Nacional Autónoma de México. Instituto de Biología, Coyoacán, 04510. México City; 2 Université de Rennes 1, Ethologie Animale et Humaine, U.M.R. 6552 - C.N.R.S, Station Biologique, 35380 Paimpont, France; 3 Institut Universitaire de France, 103 boulevard Saint-Michel 75005 Paris, France

## Abstract

Social traditions based on communication signals are widespread in birds, cetaceans and humans, but surprisingly rare in nonhuman primates known for having genetically-determined vocal repertoires. This study presents the first description of a singular case of behaviour associated with calling (placing a hand in front of the mouth while vocalizing: HFM) in black howler monkeys. We showed, first, that HFM was found only in a subset of the groups observed, at the same geographical location, and was age- and sex-specific. There was an audience effect on HFM, with highest rates when a neighbouring group was visible. HFM was non-randomly combined with audio-visual signals and always performed while roaring. High HFM rates triggered more vocal responses from group members and male neighbours, and HFM signalers temporally synchronized their behaviour in a predictable way. Finally, the positioning of the hand systematically modified the call’s auditory structure. Altogether these results support the idea that HFM is an innovated, culturally transmitted communication signal that may play a role in inter-group competition and intra-group cohesion. This study opens new lines of research about how nonhuman primates developed strategies to overcome their constraints in acoustic plasticity very early in the primate lineage.

Culture is a powerful tool for survival and an important aspect of human societies. Authors have claimed that carrying out comparative studies with animals may help understand culture’s evolutionary roots^1^. Investigations of intraspecific behavioural variability in animals show that a behavioural trait can be present in some populations or groups but absent in others; well-known examples include milk-bottle opening by tits^2^ and tool manufacturing by chimpanzees^3^. When behavioural differences between populations cannot be explained by habitat or genetic variations, they may represent socially-transmitted “traditions”^4^. Perry and his colleagues listed three criteria for traditions: 1) *intergroup variation*: the behaviour must be present in at least one social group and absent in at least one other group, 2) e*xpansion*: the number of performers must increase over time, and 3) *durability*: the behaviour must be long-lasting in the group’s repertoire^5^.

Culture and language in humans are inseparable evolutionally speaking^6^, and in nonhuman animals social traditions also play an important role in the evolution of communication, for example triggering dialectal changes in the vocalization patterns of birds^7^ and whales^8^. Given their phylogenetic proximity to humans, nonhuman primates are interesting models for comparative work. However, up to now studies have focused mainly on foraging techniques^9^, leaving traditions in communication signals by non-human primates less explored. One reason may be that non-human primates are known to be strongly constrained vocally. Vocal repertoires in those species are mostly genetically determined, and cases of social influences on acoustic plasticity are rare^10^. Nevertheless, a few cases of vocal communication traditions by apes (e.g., nest building calls in free-ranging orang-utans^11^) and Old World monkeys (e.g., human alarm call in captive Campbell’s monkeys^12^) have been reported. More examples include visual communication traditions by apes (e.g. handclasp grooming by chimpanzees and beckoning gesture by bonobos^13^,^14^,^15^,^16^,^17^) and Old World monkeys (“hand extension” by mandrills^18^). Some examples involve non-random innovated associations between auditory and visual signals in both apes (raspberry call and extended grunt associated with begging gesture in chimpanzees^19^) and Old World monkeys (ventro-ventral “rocking-embrace” signal always accompanied by lip smacking and sometimes by girney call in macaques^20^).

We investigated the potential existence of tradition based on communication signals at a more basal level of the primate phylogeny, i.e., a New World monkey species. Our study subjects were black howler monkeys (*Alouatta pigra*), well known for their long and loud call bouts, frequently accompanied by visual displays (such as aggressive body shakes)^21^,^22^. Despite their evident communicative abilities^23^, howler monkeys’ communication signals have been considered relatively inflexible^24^. However, recently we reported that black howler monkeys in Palenque National Park (southern Mexico) learn to recognize individual acoustic signatures^25^. During that playback study, we observed that some individuals sometimes placed one hand in front of their mouths while vocalizing (named *Hand-Front-Mouth* [HFM], Fig. 1).

As this HFM behaviour has not yet been reported for any howler monkey species, we here evaluate its value as a cultural behaviour and as a communication signal. In particular, (1) we documented the distribution of HFM behaviours in more or less geographically distant groups in Mexico, as well as among group members of various ages and sexes; (2) we estimated the contextual non-randomness of HFM production by studying its association with previously described communication signals (e.g., roar calls or body shakes, displays frequently used during inter-group competition^21^,^26^) and by assessing a potential audience effect on HFM rates in the neighbouring home range; (3) we tested whether HFM triggered a particular vocal response from intra- and inter-group receivers of both sexes; (4) we assessed the level of social coordination and synchronization between HFM signalers within a group; and (5) we tested whether (and how) HFM modified the acoustic structure of the associated call.

## Results

### Distribution of HFM across groups and group members

Observations focused on 19 groups and two solitary male black howler monkeys in three different geographic locations in Mexico (Table 1). However, only a subset of the groups, geographically close to each other, displayed HFM. Monkeys in six of the eight Palenque National Park groups (PNP) and in one of the seven groups living nearby in the Palenque fragmented forest (PFF) produced HFM behaviours, but none did so in the geographically distant Yucatan peninsula captive groups (YP) (Table 2). HFM production was age- and sex-specific; only adult and subadult males in these seven groups performed HFM behaviours. When HFM behaviours were observed in a group, all males of that group were systematically HFM signalers. Despite the fact that our sampling effort varied greatly among groups, we found no correlation between number of howling sequences sampled and number of sequences including HFM signals (Spearman test, N = 21, r = 0.5561, P = 0.07). Therefore, our sampling effort did not affect the presence or absence of HFM in the study groups (i.e., it is unlikely that the absence of HFM behaviour in certain groups was due simply to insufficient sampling).

### Context of HFM signaling

We calculated the individual rate per minute of HFM behaviours within howling sequences for three groups (PNP1 − N = 24 sequences, PNP2 − N = 19, and PFF2 − N = 10), which we had the opportunity to observe over long periods with an acoustic recording apparatus (see the method section for details), (Table 2). Rates did not exceed one behaviour per minute: PNP1 – M1: 0.51 ± 0.37, M2: 0.42 ± 0.40, M3: 0.11 ± 0.16; PNP2 – M1: 0.56 ± 0.50, M2: 0.50 ± 0.43, M3: 0.07 ± 0.19; PFF2 – M1: 0.97 ± 1.32; M1, 2 and 3 being different males per group). The average number of calls emitted per sequence (178 ± 120) was about 15 times higher than the average number of HFM produced per sequence (11.7 ± 10.7). Howling sequences are composed of different concatenated call types. Nevertheless, 100% of the HFM signals observed were produced at the same time as a roar call (N = 646/21170), with no HFM performed during barks (N = 0/28151), grunts (N = 0/1565) or between calls (i.e. silence gaps).

**Figure 2:**
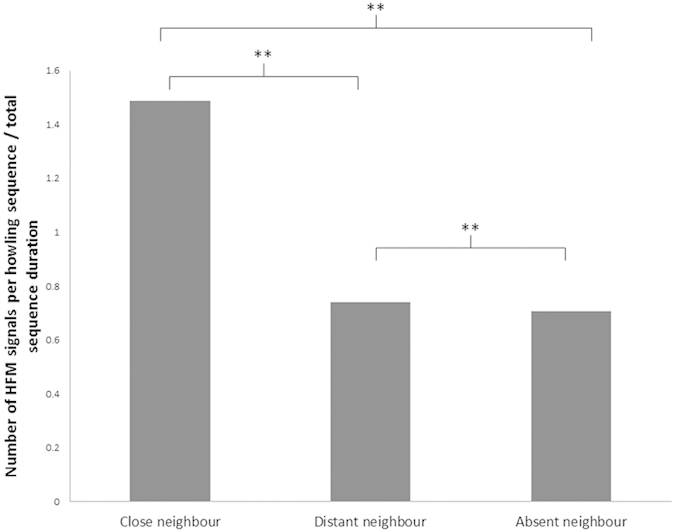
Audience effect on HFM rates, **P = 0.01; *P = 0.04; ns P > 0.05.

HFM rates increased in the presence of other groups (i.e., showed an audience effect) in the neighbouring home range (Z = 39.25, P = 0.037), independently in the three studied groups (Z = 36.53, P = 0.26) (Fig. 2). HFM rates were higher when another group was close by (i.e. auditory and visual contact, 20.3 ± 12.4% of the contexts sampled per group) than when a group was farther away (only auditory contact, P = 0.04) or when no other group was around (Post-hoc pairwise comparisons, applying Holm correction for multiple testing: P = 0.01. There was no difference in HFM rates when neighbours were far way or absent (P = 0.21).

Individuals produced more HFM behaviours when they were calling from peripheral positions (N = 24; 1.45 ± 1.08) than when they were calling from central (N = 26; 0.66 ± 0.82) positions in their home range (Z = 42.77, P = 0.005), regardless of group identity (Z = 46.76, P = 0.14). The level of group dispersion also influenced HFM rates significantly (Z = 36.2, P = 0.04, no group effect: Z = 39.2, P = 0.6); HFM rates were higher when group members were not dispersed (N = 31; 1.22 ± 1.05 HFM/min) than when they were dispersed (N = 19; 0.54 ± 0.51 HFM/min). Also, HFM occurrence was significantly associated with “body shake” displays during howling sequences (N = 50; Fisher’s exact test: P = 0.0063).

### Receivers’ vocal responses

We found a sex-dependent influence of HFM during howling sequences on the vocal activity of group members and neighbours. HFM rates were positively correlated with the number of calls produced by neighbouring males in the corresponding sequence (N = 19; Spearman test: r = 2.89; P < 0.001), but not by neighbouring females (N = 19; r = 1.6; P = 0.13). Hence, the more frequent HFM were in a sequence, the more calls were produced by neighbouring males. Within groups, no correlation was found with male (N = 50; r = 0.22; P = 0.12) or female (N = 50; r = 0.16; P = 0.25) calling rates. However, HFM rates were still related to their calling quantity, as sequences with high (i.e. above the median) HFM rates were associated with more male (N = 50; Binomial test: P < 0.001) and female (P = 0.02) calls than sequences with low (below the median) HFM rates. Interestingly the influence of HFM on calling is supported by the fact that HFM were more frequent in the first half of the vocal sequences than in the second half (N = 50; sign test P = 0.03).

### Social coordination between HFM signalers

First, a social contagion effect was found in the six groups, including two adult males producing HFM behaviours. The number of sequences in which two males produced HFM together was significantly higher than the number of sequences in which only one male produced HFM (Wilcoxon matched-paired signed-rank test: N = 6; Z = 2.301, P = 0.028).

**Figure 3:**
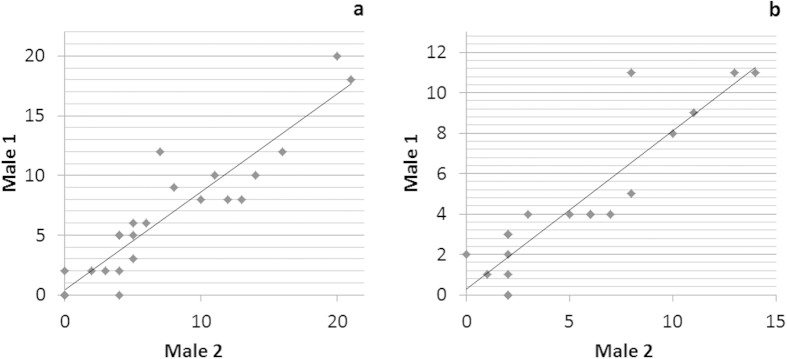
Matching of the numbers of HFM signals produced by two males when howling together ((**a)** PNP1 group, (**b**) PNP2 group).

Second, interacting individuals matched the number of HFM behaviours they performed in a given sequence. The numbers of HFM behaviours produced by the two signaling males in groups PNP1 and PNP2 (the only groups studied in detail with two adult males) were positively correlated (Spearman tests, PNP1: N = 24, r = 0.92, P < 0.001; PN2: N = 19, r = 0.91, P < 0.001) (Fig. 3).

**Figure 4:**
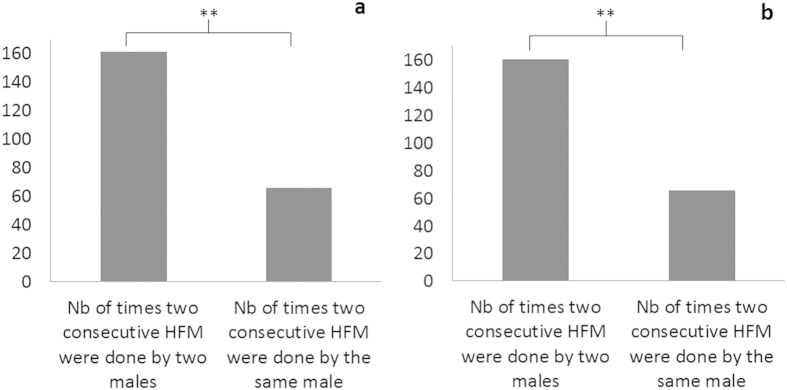
Turn-taking in HFM signaling ((**a**) PNP1 group, (**b**) PNP2 group), **P < 0.001.

Third, our data revealed a turn-taking pattern in HFM production. Male group members synchronized their productions by alternating HFM rather than repeating their own production (Chi-squared tests, PNP1: N = 24, X = 39.7, P < 0.001; PNP2: N = 19, Z = 9.9, P < 0.001, Fig. 4).

### Acoustic changes associated with HFM

Discriminant Function Analyses (DFA) showed that HFM predictably modified the acoustic structure of roar calls. The percentage of correct classification of the two categories (roar with and without HFM) was much higher than expected by chance (i.e. 50%), as 89% of the calls were correctly assigned to their category. MANOVA test confirmed a significant difference between calls with and calls without HFM (F_20,57_ = 14.59, P < 0.001, Wilk’s λ = 0.04). The cross-validated DFA, using random calls (N = 20 calls, 10 calls per category) to build the model, confirmed the correct classification (85%). The acoustic parameter that contributed the most to the classification (more than 3 times more than all other acoustic parameters) was the 3rd Quartile Frequency (roar with HFM = 750+/− 147 Hz; roar without HFM = 862+/− 98 Hz).

## Discussion

According to Perry and his colleagues’ definition^5^, HFM behaviour by black howler monkeys qualifies as a tradition. First, HFM has never been reported in other New Wold primate species, was found only in a subset of the groups studied, and was age- and sex-specific, confirming that it is not widespread in this species. Second, our data suggest expansion and social transmission of HFM: (1) when HFM was observed in a group, all the adult and subadult male members performed this behaviour; (2) HFM was present only in groups geographically close to one another. Neighbouring groups in the Palenque population are genetically more dissimilar than more distant groups^27^,^28^,^29^. This is important because it supports a cultural (non-genetically based) transmission. Third, HFM is durable: intermittent monitoring between 2011 and 2014 confirmed that this behaviour was present throughout this period. However, a longitudinal study with continuous observations is now needed to determine how the signal spreads among group members and groups.

Traditions usually concern subsistence activities, body care, or social behaviours, and they sometimes have no apparent utility or purpose, as found in chimpanzees^30^. Here, we believe HFM to be a communication signal. First, HFM behaviours are involved in non-random combinations with other communication signals. HFM are associated only with roar calls and alternate with body-shake displays. Second, HFM are produced more frequently when group members are spatially cohesive, supporting a social context of production. Third, HFM signalers coordinate their production of HFM by performing HFM synchronously and at similar rates when together. Fourth, individuals took turns while producing HFM behaviours. Turn-taking is one of the characteristic interaction patterns found in communication^31^,^32^, typically playing a role in the maintenance of socio-spatial cohesion^5^. Fifth, HFM has an impact on the behaviours of receivers, which is an essential criterion of communication^33^. It increased the vocal activity of receivers in a sex- and social-dependent way (i.e. male and female group members as well as male neighbours). Finally, an audience effect on HFM rates was found, as HFM are most frequent during inter-group encounters.

The social function of HFM signals remains an open question, deserving more detailed observations of what occurs in both emitters and receivers when a male is *vs* is not performing HFM. However, we believe that the HFM signal plays a role in both inter-group competition and intra-group cohesion. The former conclusion is drawn from its association with agonistic displays (body/branch shakes) and roar calls, which are known to play a key role during agonistic inter-group encounters^21^,^34^. It is also drawn from the fact that HFM rates were correlated with male neighbour calling effort and audience effect at the border of the home range. The latter conclusion is drawn from the observed group spatial cohesiveness, the fact that male and female group members join the howling event more frequently, and the coordination between males from the same group associated with signaling.

How HFM signals should qualify is another still open question. Two plausible hypotheses can be formulated at this stage. One hypothesis is that HFM is only an auditory signal. Placing the hand in front of the mouth would thus be a postural innovation associated with calling. This is supported by the fact that HFM modifies the acoustic structure of the call produced. Interestingly, the main acoustic parameters affected tend to show that HFM makes the emitter’s voice appear deeper. It is well known that lower-pitched calls travel farther and are associated with males of larger body size^35^. Again, this would support an inter-group competition function. Cases of nonhuman primates using behaviours to modify their voices are extremely rare. A study of orang-utans showed the ability of individuals to modify acoustic parameters during kiss-squeak using leaves as a tool or putting a hand to the mouth, which the authors suggested could convey (falsified) information to the predator about their body size^36^. Another hypothesis is that HFM is a multimodal (audio-visual) innovated signal and that the arm position conveys extra information not conveyed by the call. While it seems obvious that the arm position of the emitter is visible both to group members and to close neighbours, we do not have access to data showing that receivers actually visually pay attention to the posture. Further experiments and observations are needed to sort these two hypotheses.

In conclusion, we showed here a rare case of tradition of a new behaviour associated with calling in nonhuman primates. The questions of whether this is an uni- (auditory) or multi-modal (audio-visual) signal, and how this behaviour appeared and spread, remain open, deserving further investigations. Our results suggest that HFM behaviour is more than simply a fashion without apparent function, but rather that it has some role in intra-group and inter-group communication. Interestingly, the fact that HFM behaviour was found in New World monkeys suggests that different strategies to compensate for non-human primates’ limited acoustic plasticity may have emerged very early in that lineage^37^,^38^.

## Methods

### Study sites and groups

Observations focused on 19 groups and two isolated adult male black howler monkeys (*Alouatta pigra*) (see Table 1 for group characteristics and sampling efforts) in three different geographical areas:

   (1)  Palenque National Park (PNP): here we observed eight free-ranging social groups with adjacent territories at Palenque National Park, Mexico. This 17.7 km^2^ park includes 6.0 km^2^ of primary tropical continuous rain forest where these groups live^39^. These groups included 1 to 3 males, 1 to 3 females, and their offspring.

   (2)  Palenque fragmented forest (PFF): here we studied seven free-ranging social groups, each one living in its own fragmented forest patch, 10.3 ± 8.4 km from PNP. These patches included agricultural lands, urban areas with dispersed trees, and dense secondary forest. These groups included 1 or 2 males, 1 to 3 females, and their offspring.

   (3) Yucatan peninsula (YP): here we studied four social groups (composed of 1 male, 1 to 2 females and their offspring) and two isolated individuals in captivity (Xcaret Park), 760 km from Palenque in the Yucatan peninsula of Mexico. All adult subjects were wild-born on the Yucatan peninsula (they were caught in the same region after the destruction of their habitat by a hurricane and housed according to the original group composition). Three social groups were housed in 3 × 3 × 6 m cages and the individual monkeys in 3 × 2 × 3 m cages, enriched with perches for climbing (all of them had auditory contact with neighbours and all but one also had visual contact). The fourth group (YP4), visually and auditorily isolated, lived on an island (8 m diameter). Animals were fed twice a day (6 am and 6 pm with fruit and kibble). Water was available *ad libitum*.

Our research complied with protocols of the Animal Care Committee of Universidad Nacional Autonoma de Mexico and adhered to the legal requirements of Mexico. Protocols were approved by Direccion General de Vida Silvestre (SEMARNAT), permit SGPA/DGVS/00692/08.

### Observations, acoustic recordings and statistical analyses

#### HFM distribution across groups and group members

To evaluate the distribution of HFM across groups and group members, we observed all subjects available, i.e. 19 social groups and two isolated individuals. HFM never occurred outside the long and loud howling sequences in any of the individuals observed. To assess its distribution, a single observer (MBJ) scored, for each of the 183 howling sequences observed (Table 1), the identity of the individuals who performed at least one HFM using the one/zero sampling method^40^. This was used to calculate individual HFM contribution scores (number of sequences during which an individual displayed at least one HFM/number of vocal sequences including this individual*100).

#### Context of HFM signaling

The subsequent analyses (i.e. context of HFM signaling, receivers’ vocal responses, acoustic changes associated with HFM) were done with the data from only three of the groups presenting HFM behaviours (i.e. PNP1, PNP2 and PFF2), because those groups could be observed in more detail and over longer periods with an acoustic recording apparatus. A howling sequence (defined by Kitchen^41^ as a long-lasting howling bout with concatenation of different call types, i.e., roars, barks, and grunts) is composed of different calls produced by different callers. Howling sequences occurred about once or twice a day per group with a within-sequence inter-call interval of 0.54 sec +/− 0.07 sec. HFM behaviours always occurred while a signaler was producing a call. Acoustic recordings were used to ensure the classification of call types associated with HFM. Calls were classified as barks, roars and grunts (for definitions see^42^,^43^). The recording apparatus included a directional microphone (SONY ECM-672) and a tie microphone (EUROPSONIC ECM 104), connected to a digital audio recorder (MARANTZ PMD670) (Sample rate 44.1 kHz, resolution 16 bits, WAV format). The first track was used to record monkey calls and the second track was used to record caller and HFM signaler identities during each howling event. All recordings were made at comparable distances from callers (20 to 30 m). For these three groups we scored the identity of each signaler and each caller during all the howling sequences, using the all-occurrence sampling method^31^.

The context of signaling by these three groups was evaluated by recording during each howling sequence:

- the type of audience in the neighbouring home range: absence of a neighbouring group, a neighbouring group in the distance (only audible), or a neighbouring group nearby (audible and visible);

- the position of the focus group on its home rage when a howling sequence started: peripheral (site where previous group encounters were observed) or central (site where no group encounters had been observed previously);

- dispersion of group members: not dispersed or dispersed (when more than 50% of the group members were distributed over an area above 25 m^2^)^44^;

- other behaviours: when at least one “body-shake” display, which is a discomfort and agonistic signal^26^,^45^, occurred during a howling sequence.

Using a Binomial Generalized Linear Model, we tested the influence of howling context (neighbour absent, distant, or close), group position (central, peripheral), dispersion (dispersed or not) and identity on HFM rates (number of HFM behaviours per howling sequence/total sequence duration in minutes). A Fisher exact test was used to estimate statistically the contextual association between HFM signals and body shakes.

#### Receivers’ vocal responses

For all sequences in PNP1, PNP2 and PFF2 groups, we calculated the rate (per minute) of HFM production, the number of calls emitted per male and per female within the focal group and in the neighbouring group at the time of encounters. Then, Spearman tests were done to explore the relationships between those measures. When no correlation was found, we also used Binomial tests to compare the number of calls emitted in sequences with high (above the median) vs low (below the median) HFM rates.

#### Social coordination between HFM signalers

First, to assess the level of social contagion among HFM signalers, based on data for all the groups (i.e. PNP1, PNP2) producing HFM signals and including two adult males, we compared, using a Wilcoxon test, the percentages of sequences in which one *versus* two signalers were observed. Second, based on data for the groups sampled using the all-occurrence regime, Spearman correlation tests compared the numbers of HFM signals per sequence produced by the two contributing males. This analysis (and the following) was done with PNP1 and PNP2, as PFF2 was a single male group. Third, we evaluated adaptation between synchronization of signalers’ behaviour and a turn-taking pattern throughout a given sequence by comparing, using Chi-squared tests, the number of times a given male repeated his own HFM twice in a row with the number of times two males alternated HFM.

#### Acoustic changes associated with HFM

Spectrograms were generated using Raven bioacoustics software (v. 1.2, Cornell Bioacoustics Laboratory), with a Fast Fourier transformation (FFT) and a time window of 256 points. In order to estimate if HFM influenced the acoustic structure of the call produced, we measured the same eight representative acoustic parameters as in Briseño Jaramillo *et al.*^25^ (see definitions in Table 3): Lowest frequency (Low Freq, Hz), Highest frequency (High Freq, Hz), 1st and 3rd quartile frequency (Q1freq and Q3freq, Hz), Aggregation of entropy (Aggr Entropy, Hz), 90% bandwidth (BW 90%, Hz), Total duration (s), and Energy (dB). We randomly selected a sample of 50 roar calls with HFM and 50 calls without HFM, produced by five males (N = 10 calls per male for each category) from the three groups. We never selected more than three calls per sequence to limit pseudoreplication.

For each acoustic parameter measured, normality was confirmed with Shapiro tests (p > 0.05 in all cases). We also confirmed, by visually inspecting box plots, that the variances of our parameters were homogeneous. Then, we transformed the eight acoustic variables into a set of non-correlated components using Principal Component Analyses (PCA). To determine the number of relevant components (PC) for each call type, we used the Kaiser-Guttman criterion (keeping only PCs with eigenvalues > 1). We ran a MANOVA analysis using these components to assess the contribution of each acoustic parameter in the discrimination between calls. To estimate the reliability of this discrimination, a first Discriminant Function Analysis (DFA) was performed on the entire data set, and then a second more conservative cross-validated DFA was done. For this second DFA, a subset of 20% of the calls (N = 10/calls per category) was randomly used for establishing the model. The remaining calls were then used to test the classification (as in Crockford & Boesh^46^ and Briseño Jaramillo *et al.*^25^).

## Additional Information

**How to cite this article**: Briseño-Jaramillo, M. *et al.* Behavioural innovation and cultural transmission of communication signal in black howler monkeys. *Sci. Rep.* **5**, 13400; doi: 10.1038/srep13400 (2015).

## Figures and Tables

**Figure 1:**
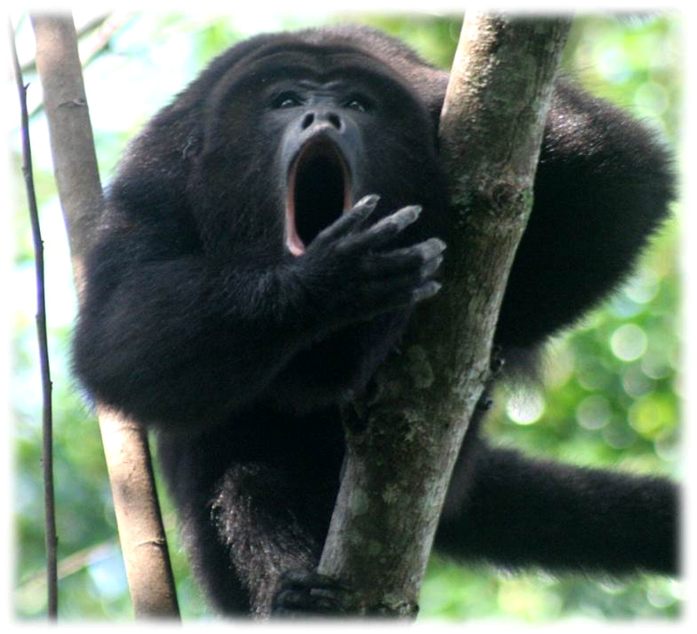
A black howler monkey placing his hand in front of his mouth while vocalizing. Photo: Eloise Chailleux.

**Table 1:** Characteristics of the 21 groups of black howler monkeys studied in three geographical locations.

Geographical location	Study group and isolate individuals	Group composition (adult males/adult females/subadult males/subadult females/offspring)	Number of contact hours (observation periods)	Number of sampled sequences
Palenque National Park (PNP)	PNP1	3/3/0/1/3	291 h (Feb. - Apr. 2012, Feb. 2014)	24
	PNP2	2/1/1/0/4	297 h (Apr. - Jun. 2012, Feb. 2014)	19
	PNP3	2/2/1/0/1	300 h (Jun. - May. 2012, Feb. 2014)	29
	PNP4	1/2/0/1/1	92 h (Feb. - Mar. 2014)	7
	PNP5	2/2/1/0/1	55 h (Jun. 2012, Mar 2013, Feb. 2014)	5
	PNP6	2/3/1/1/1	57 h (Jun. 2012, Mar 2013, Feb. 2014)	7
	PNP7	2/3/1/1/3	60 h (Jun. 2012, Mar 2013, Feb. 2014)	6
	PNP8	2/3/0/0/4	4 h (Jun. 2012, Mar 2013, Feb. 2014)	5
Palenque fragmented forest (PFF)	PFF1	1/2/1/0/2	48 h (Feb. - Mar. 2014)	9
	PFF2	1/1/0/0/2	20 h (Feb. 2014)	10
	PFF3	2/2/1/1/3	25 h (Feb. - May 2014)	13
	PFF4	1/2/0/0/3	4 h (Mar. 2013, Feb. 2014)	4
	PFF5	2/2/0/1/1	4 h (Mar. 2013, Feb. 2014)	3
	PFF6	2/3/0/0/1	3 h (Mar. 2013, Feb. 2014)	3
	PFF7	2/2/0/0/4	2 h (Mar. 2013, Feb. 2014)	3
Yucatan peninsula (YP)	YP1	1/2/1/1/4	156 h (Nov. 2011, Aug. - Sep 2012)	6
	YP2	1/1/0/0/2	156 h (Nov. 2011, Aug. - Sep 2012)	7
	YP3	1/1/0/0/2	156 h (Nov. 2011, Aug. - Sep 2012)	6
	YP4	1/1/0/0/2	156 h (Nov. 2011, Aug. - Sep 2012)	5
	YP5	1/0/0/0/0	156 h (Nov. 2011, Aug. - Sep 2012)	6
	YP6	1/0/0/0/0	156 h (Nov. 2011, Aug. - Sep 2012)	6

**Table 2:** Individual contribution scores to HFM signaling.

Group name	Adult male			Subadult male	Adult female			Subadult female
	***M1***	***M2***	***M3***	***SM1***	***F1***	***F2***	***F3***	***SF1***
PNP1	87.5	79.2	29.2		0	0	0	0
PNP2	100	84.2		47.4	0			
PNP3	0	0		0	0	0		
PNP4	0				0	0		0
PNP5	80	80		60	0	0		
PNP6	85.7	71.4		57.1	0	0	0	0
PNP7	83.3	66.7		66.7	0	0	0	0
PNP8	60	40			0	0	0	
PFF1	0			0	0	0		
PFF2	40				0			
PFF3	0	0		0	0	0		0
PFF4	0				0	0		
PFF5	0	0			0	0		0
PFF6	0	0			0	0	0	
PFF7	0	0			0	0		
YP1	0			0	0	0		0
YP2	0				0			0
YP3	0				0			0
YP4	0				0		0	0
YP5	0							
YP6	0							

In each cell: number of sequences during which an individual displayed at least one HFM/number of sequences vocally emitted by this individual*100.

**Table 3:** Acoustic measurement definitions.

	Definition
Lowest frequency (Low Freq, Hz)	The lower frequency bound of the call.
Highest frequency (High Freq, Hz)	The upper frequency bound of the call.
1st Quartile Frequency (Q1fre, Hz)	The frequency that divides the call into two frequency intervals containing respectively 25% and 75% of the energy distribution.
3rd Quartile Frequency (Q3fre, Hz)	The frequency that divides the call into two frequency intervals containing respectively 75% and 25% of the energy distribution.
Aggregation of entropy (Aggr Entropy, Hz)	The degree of disorder (i.e. noisiness) in the call.
90% bandwidth (BW 90%, Hz)	Amplitude between the frequencies measured at 5 and 95% of the energy distribution.
Total Duration (s)	The temporal difference between the beginning and the end of the call.
Energy (dB)	The total energy in the call.
